# Astragalus Polysaccharide Exerts a Neuroprotective Effect in a Demyelination Model by Alleviating Oxidative Stress

**DOI:** 10.1002/fsn3.72579

**Published:** 2026-11-06

**Authors:** Yijiao Long, Lihui Wang, Baohua Hou

**Affiliations:** ^1^ School of Medicine Henan Polytechnic University Jiaozuo China; ^2^ Jiaozuo Key Laboratory for Huaiyao Comprehensive Development Henan Polytechnic University Jiaozuo China

**Keywords:** Astragalus polysaccharide, demyelination, neuroprotection, oxidative stress

## Abstract

Multiple sclerosis (MS) is an autoimmune disease of the central nervous system characterized by progressive demyelination and neurodegenerative damage. The accumulation of reactive oxygen species (ROS) and the resulting oxidative stress are considered key contributors to myelin injury and disease progression. Astragalus polysaccharide (APS), a water‐soluble polysaccharide, exhibits multiple pharmacological activities, including immunomodulatory, anti‐inflammatory, antioxidant, and anti‐apoptotic effects. This study aimed to investigate the therapeutic effects of APS in MS and to explore its potential mechanisms. In vivo, APS was administered intraperitoneally at 50 mg/kg/day in a cuprizone‐induced demyelinating mouse model, and its effects were evaluated by immunofluorescence, immunohistochemistry, and Western blotting. In vitro, the regulatory effects of APS on oxidative stress in oligodendrocytes were assessed by immunofluorescence and Western blotting in an H_2_O_2_‐induced oligodendrocyte injury model. APS significantly ameliorated myelin damage and apoptosis in vivo by inhibiting oxidative stress and attenuated oxidative stress and apoptosis in oligodendrocytes in vitro, thereby exerting neuroprotective effects. These findings indicate that APS alleviates demyelination by regulating oxidative stress, suggesting that it may represent a promising therapeutic option for the treatment of multiple sclerosis.

## Graphical abstract

Cuprizone‐induced demyelination is associated with dysregulation of the NRF2/HO‐1 axis and ROS accumulation. APS treatment upregulates NRF2, inhibits HO‐1 and reduces ROS, thereby alleviating demyelination.

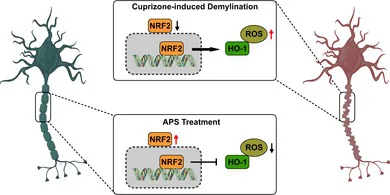

## 1 Introduction

A chronic autoimmune disorder affecting the central nervous system (CNS) is multiple sclerosis (MS), with pathological features dominated by ongoing demyelination (Khan et al. 2023). Recent estimates suggest that MS affects around 2.8 million individuals across the globe (Walton et al. 2020). To date, the pathogenesis of MS remains incompletely understood, and effective treatments are limited, which severely impacts patient function and quality of life. Therefore, the identification of novel therapeutic agents that effectively inhibit demyelination with better reliability and security features is of critical importance for MS treatment.

Oligodendrocytes are essential for the development of CNS myelin. Mature oligodendrocytes (OLs) develop from oligodendrocyte precursor cells (OPCs), which extend processes to envelop axons, ultimately forming compact multilamellar myelin sheaths (Stadelmann et al. 2019; Chang et al. 2016). Oligodendrocytes are more vulnerable to neurotoxic and excitotoxic damage because of their energy‐demanding physiology and increased metabolic turnover. Consequently, oligodendrocyte dysfunction features prominently in diverse neurological disorders, notably multiple sclerosis, schizophrenia, and Alzheimer's disease (Kuhn et al. 2019). Thus, safeguarding oligodendrocytes is regarded as a valuable therapeutic approach for inhibiting demyelination.

The pathogenesis of MS is highly complex, but mounting data indicates that oxidative stress constitutes a major contributor to its pathogenesis (Ohl et al. 2016; Gilgun‐Sherki et al. 2004). Free radicals and related molecules generate reactive oxygen species (ROS) and serve as key signaling molecules in oxidative damage to cellular components. Oxidative stress occurs when the generation of intracellular ROS and other oxidants overwhelms the cellular antioxidant system, thereby leading to oxidative impairment in lipids, proteins, and DNA (Eruslanov and Kusmartsev 2010). In MS and its extensively employed animal counterpart, experimental autoimmune encephalomyelitis (EAE), ROS have been identified as critical factors serving a critical function in the pathogenesis of demyelination and axonal injury, thereby contributing to oxidative stress (Gilgun‐Sherki et al. 2004). Nuclear factor erythroid 2‐related factor 2, abbreviated as Nrf2, is a pivotal antioxidant defense‐related transcription factor and the maintenance of cellular redox homeostasis (Davinelli et al. 2025). Nrf2 pathway activation is known to effectively attenuate oligodendrocyte loss, neuroinflammation, and axonal damage in MS (Nellessen et al. 2020). Heme oxygenase‐1 (HO‐1) represents a downstream effector of Nrf2 and is upregulated in Alzheimer's disease patients and MS models (Premkumar et al. 1995; Moezzi et al. 2022). Therefore, targeting oxidative stress represents a promising therapeutic strategy for MS.

One of Astragalus membranaceus's primary active ingredients is Astragalus polysaccharide (APS), a traditional Chinese medicinal herb, and possesses a complex structure with diverse pharmacological activities (Shi and Ma 2024). APS is a water‐soluble polysaccharide whose primary monosaccharide component is glucan, with minor modifications by galactose and arabinose (Cai et al. 2023). APS exhibits antiviral, antitumor, anti‐inflammatory, antioxidant, and immunomodulatory activities (Tang and Huang 2022). APS has been reported to trigger the Nrf2 pathway in APP/PS1 mice, reducing apoptosis and Aβ accumulation, thereby improving cognitive function in Alzheimer's disease (Qin et al. 2020). Additionally, APS promotes autophagy in PC12 cells through the AKT/mTOR/PI3K pathway, exerting anti‐Parkinson effects (Tan et al. 2020). Moreover, APS can restore tight junction proteins and alleviate the destruction of the blood–brain barrier (BBB) (Yao et al. 2026). However, studies on the regulatory effect of APS in MS remain limited.

Therefore, the goal of the current study was to elucidate the underlying mechanisms and therapeutic effects of APS in MS, thereby providing a new strategy for the development of MS therapeutics.

## 2 Materials and Methods

### 2.1 Chemicals and Reagents

Astragalus polysaccharide (APS) (89250‐26‐0, Macklin, Shanghai, China), cuprizone (CPZ) (370‐81‐0, Macklin), ML385 (HY‐100523, MedChemExpress), and hydrogen peroxide (H_2_O_2_, 3% solution) (7722‐84‐1, Macklin) were used in this study.

### 2.2 Animals and Treatments

Male C57BL/6 mice (20–24 g) were obtained from Beijing Vital River Laboratory Animal Technology Co. Ltd. Every experiment conducted in animals was authorized by the Ethical Committee of Henan Polytechnic University. Mice were kept in controlled environments, having unrestricted access to water and food (a 12‐h light/dark cycle was employed, with temperature set at 22°C ± 2°C and humidity at 50% ± 5%). After a 1‐week acclimation period, the mice were randomly allocated to three groups (*n* = 10 per group): control, CPZ, and CPZ + APS. Animals in the control group were administered normal saline via intraperitoneal injection. CPZ (300 mg/kg) was administered to the animals in the experimental group via oral gavage twice daily for two consecutive weeks to induce demyelination. Before CPZ was administered, mice in the CPZ + APS group were given APS (50 mg/kg, i.p.) once per day for five consecutive days. From day six onward, these mice received CPZ (300 mg/kg, gavage, twice daily) concurrently with APS (50 mg/kg, i.p., once daily) for 2 weeks. After the treatment course, mice were anesthetized with tribromoethanol, underwent myocardial perfusion, and collected brain tissue specimens for further research. This study used G*Power 3.1 to perform an a priori sample size estimation. The statistical test employed was a one‐way fixed‐effects analysis of variance (three groups, two‐tailed *α* = 0.05, target power of 80%). Based on the effect size of *f* = 0.6400195 estimated from the pilot study, 9 mice were required per group; after adjusting for an anticipated dropout rate of approximately 10%, 10 mice were enrolled in each group (Festing 2018).

### 2.3 Cell Culture and Cell Viability Assay

OLN93 oligodendrocytes (FH1317, FUHENG BIOLOGY, China) were maintained in DMEM (L110KJ, BasalMedia, China) enriched with 1% penicillin‐streptomycin (BL505A, Biosharp) and 10% fetal bovine serum (FBS, A5256701, Gibco, São Paulo, Brazil), with incubation carried out at 37°C inside a humidified chamber supplied with 5% CO_2_.

A cellular model of demyelination was constructed with H_2_O_2_‐induced OLN93 oligodendrocyte injury (Zha et al. 2022). The Cell Counting Kit‐8 (CCK‐8, MC0301‐500, Kermey, Zhengzhou, Henan, China) assay was used to estimate the ideal H_2_O_2_ and APS concentrations. 96‐well plates were seeded with 100 μL of cells per well. Following 24 h of incubation, the culture medium was replaced with serum‐free formulations supplemented with H_2_O_2_ across a range of concentrations (200, 400, and 800 μM) for 24 h. After that, cells were treated for 30–60 min at 37°C with CCK‐8 reagent. Absorbance readings at 450 nm were acquired via a microplate reader (SpectraMax Mini, Molecular Devices, Shanghai, China), allowing viability values to be computed.

Culture was initiated by seeding cells in 96‐well plates. Three groups of cells were randomly assigned: (1) a control group that was cultivated normally; (2) H_2_O_2_ group, treated with 400 μM H_2_O_2_ in medium devoid of serum for 24 h; and (3) H_2_O_2_ + APS group, pretreated with APS at indicated concentrations (12.5, 25, 50, or 100 μg/mL) for 2 h, then handled with 400 μM H_2_O_2_ and APS at the same concentrations for 24 h. Subsequently, after adding the CCK‐8 reagent, cells endured incubation for 30–60 min at 37°C. Absorbance at 450 nm was subsequently measured to ascertain cell viability.

Cells were plated into 6‐well and 96‐well plates and randomly assigned to one of four experimental groups. The untreated control group received no additives. The H_2_O_2_ group was incubated for 24 h in serum‐free DMEM supplemented with 400 μM H_2_O_2_. The H_2_O_2_ + APS group was maintained under the same conditions but with the addition of 25 μg/mL APS. For the fourth group, 5 μM ML385 was included along with H_2_O_2_ and APS to block Nrf2, thereby enabling us to explore the protective mechanism of APS against oligodendrocyte injury.

### 2.4 Detection of Intracellular Reactive Oxygen Species (ROS)

Quantification of intracellular ROS was carried out with the ROS Detection Kit (S0035S, Beyotime Biotechnology, China), adhering to procedures provided by the manufacturer. Cells from distinct treatment conditions were incubated at 37°C for 30 min with 5 μM CM‐H2DCFDA, subsequently washed thrice with PBS. Next, a fluorescent microscope (Leica DMi8, Leica, Germany) was adopted to take fluorescence images.

### 2.5 Immunohistochemistry

Following deep anesthesia, perfusion via the transcardial route was carried out on mice employing saline, after which 4% paraformaldehyde (PFA, BL539A, Biosharp) was administered. Upon completion of fixation employing 4% PFA, the brain tissues were processed for paraffin embedding. Brain sections were then processed for immunohistochemistry. The procedure included the following steps: sections were baked at 60°C for dewaxing, rehydrated through a graded ethanol series, and treated with 0.3% Triton X‐100 (T8200, Solarbio, Beijing, China) to increase permeability; incubation with 3% hydrogen peroxide prepared in methanol was performed on sections to eliminate endogenous peroxidase. Citrate buffer (BL619A, Biosharp) was applied to sections for antigen retrieval. Sections were first heated to boiling in a microwave at high power (800–1000 W), then maintained at medium‐low power (20%–30% power) for an additional 10 min. After blocking the sections with 10% goat serum (C0265, Beyotime Biotechnology, Shanghai, China), primary antibodies were then applied, with incubation lasting overnight at 4°C. On the following day, sections were allowed to react with secondary antibodies (1:500, E030110, E030120, EarthOx, Beijing, China) for 1 h at ambient temperature, followed by a DAB staining solution (ZLI‐9019, ZSGB‐BIO, Beijing, China). The principal antibody reagents employed were: anti‐MBP (1:500, GTX133108, GeneTex, USA), anti‐Nrf2 (1:500, 16396‐1‐AP, Proteintech, Wuhan, Hubei, China), anti‐HO‐1/HMOX1 (1:500, 10701‐1‐AP, Proteintech). The Olympus AH‐2 light microscope (Olympus, Tokyo, Japan) was used to take the images, and the integrated optical density (IOD) of targeted regions was quantified utilizing ImageJ software (v1.51, National Institutes of Health, Bethesda, MD, USA).

### 2.6 Immunofluorescence

For tissue samples, after being deparaffinized, hydrated, and permeabilized, sections underwent antigen repair. Following a blocking step with 10% normal goat serum, primary antibodies were added to samples. Incubation took place overnight at 4°C. After that, a one‐hour incubation at room temperature with CY3 or DyLight 488 secondary antibody was performed on sections (1:500, E031620, E032210, EarthOx). Lastly, nuclei were stained using 4′,6‐diamidino‐2‐phenylindole (DAPI, D9542, Sigma‐Aldrich). A Leica fluorescence microscope served to obtain images. For cultured cells, after the indicated treatments, the supernatant was discarded, and PBS was used to rinse the cells. 4% PFA was used to fix the cells, washed in PBS three times, and permeabilized with 0.3% Triton X‐100. 10% goat serum was applied for cell blocking treatment following further washing, and they were then treated with primary antibodies for an overnight at 4°C. Three washes using PBS were performed, after which cells were incubated with fluorescent secondary antibody for 1 h. Finally, cell nuclei were stained using the DAPI staining reagent, and images were captured using a Leica fluorescence microscope. The major antibodies utilized were as follows: anti‐Olig2 (1:500, GTX132732, GeneTex), anti‐PDGFRα (1:500, AF1062, R&D Systems, USA), anti‐Nrf2 (1:500, 16396‐1‐AP, Proteintech), anti‐HO‐1/HMOX1 (1:500, 10701‐1‐AP, Proteintech). Fluorescence relative intensity values were quantitatively analyzed by means of ImageJ analytical software.

### 2.7 Western Blot

Phosphatase (BL615A, biosharp) and protease (BL612A, biosharp) inhibitors were added to RIPA lysis buffer (BL504A, Biosharp) to lyse tissues and oligodendrocytes for 30 min. The fractions of brain tissue homogenates were separated under low‐temperature conditions at 12,000 rpm for 15 min. The resultant supernatant was gathered for additional analysis. For cultured cells, the lysates were collected by scraping and used for subsequent experiments. Protein concentration quantification was conducted using the BCA protein detection kit (P0006, Beyotime Biotechnology). Following sodium dodecyl sulfate‐polyacrylamide gel electrophoretic separation, 30 μg of protein samples were transferred onto polyvinylidene fluoride membranes (IPVH00010, Millipore, Darmstadt, Germany). Next, as previously mentioned, membranes were subjected to blocking treatment with quick blocking buffer for 10 min (Liu et al. 2023). Overnight incubation at 4°C was performed on membranes using primary antibodies. Following this, the membranes were incubated with HRP‐labeled secondary antibodies (1:50,000, E030110, E030120, EarthOx) for 2 h at ambient temperature. An enhanced chemiluminescence (ECL) detection kit (BL520B, Biosharp, China) was used to see protein bands. The grayscale intensity of target protein bands was quantitatively analyzed via ImageJ analytical software. The following primary antibodies were used: anti‐β‐actin (1:20,000, 66009‐1‐Ig, Proteintech), anti‐MBP (1:5000, GTX133108, GeneTex), anti‐Bax (1:1000, WL01637, Wanleibio, Beijing, China), anti‐HO‐1 (1:1000, 10701‐1‐AP, Proteintech), anti‐Caspase 3 (1:1000, CPA1139, Cohesion Biosciences, UK), anti‐Nrf2 (1:1000, 16396‐1‐AP, Proteintech).

### 2.8 Statistical Analysis

All experimental data were presented as the mean ± standard error of the mean (SEM) values. GraphPad Prism (version 7, GraphPad Software Inc., San Diego, CA, USA) was utilized to conduct statistical analyses. Statistical differences among multiple experimental groups were analyzed via one‐way analysis of variance (ANOVA). Differences with *p* < 0.05 were considered statistically significant, and exact *p* values are annotated directly on the respective figures.

## 3 Results

### 3.1 Astragalus Polysaccharide Ameliorates Oligodendrocyte Reduction in the Demyelination Model

Oligodendrocytes provide metabolic and functional support to neurons and serve a crucial function in the central nervous system (Tiane et al. 2019). The loss or death of oligodendrocytes is a hallmark of demyelinating diseases (de Almeida et al. 2023). Immunofluorescence labeling was used to evaluate the expression of the oligodendrocyte marker Olig2. As shown in Figure 1, Olig2 expression was markedly reduced in the corpus callosum, hippocampus, and cortex of CPZ‐treated mice compared to controls (all *p* < 0.05). In contrast, Olig2 expression was restored in the CPZ + APS group (all *p* < 0.05). The above findings show that APS treatment attenuates the loss of oligodendrocytes in the CPZ‐induced demyelination model.

**FIGURE 1:**
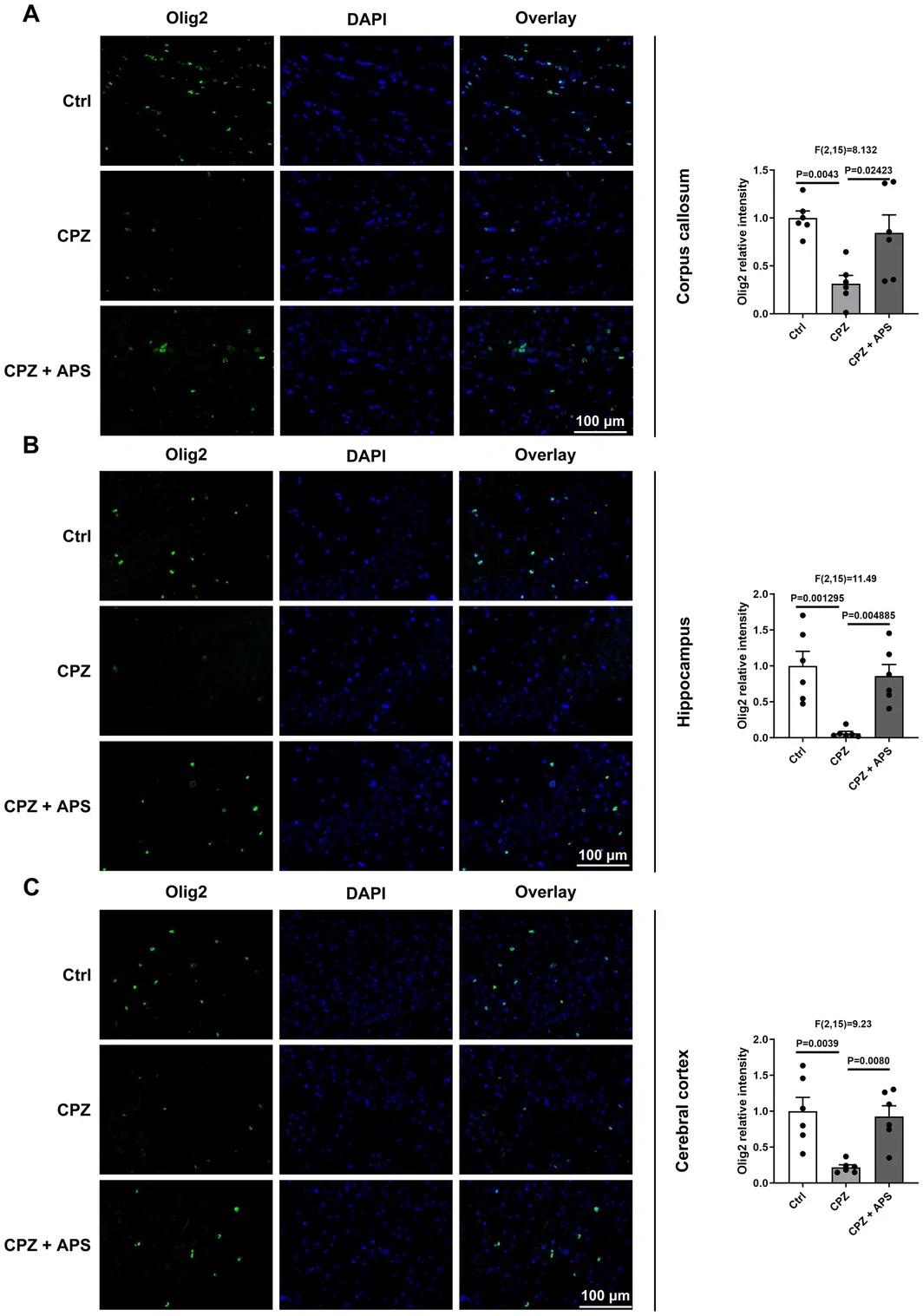
APS attenuates oligodendrocyte loss in the CPZ‐induced demyelination model. (A–C) Immunofluorescence image analysis and statistical analysis of Olig2 expression in the corpus callosum, hippocampus, and cerebral cortex were performed in different groups of mice. Scale bar = 100 μm. Statistical results are illustrated as mean ± SEM, *n* = 6. DAPI, 4′,6‐diamidino‐2‐phenylindole.

### 3.2 Astragalus Polysaccharide Ameliorates Oligodendrocyte Precursor Cell Depletion in the Demyelination Model

Oligodendrocyte precursor cells differentiate into mature myelinating oligodendrocytes (Spaas et al. 2021). Immunofluorescence analysis was carried out to evaluate the expression levels of the oligodendrocyte precursor marker PDGFRα. As shown in Figure 2, PDGFRα expression was obviously downregulated in the corpus callosum, hippocampus, and cortex of CPZ‐treated mice compared to controls, whereas APS treatment significantly restored its expression (all *p* < 0.05). These findings demonstrate that APS treatment attenuates the depletion of oligodendrocyte precursor cells in the CPZ‐induced demyelination model.

**FIGURE 2:**
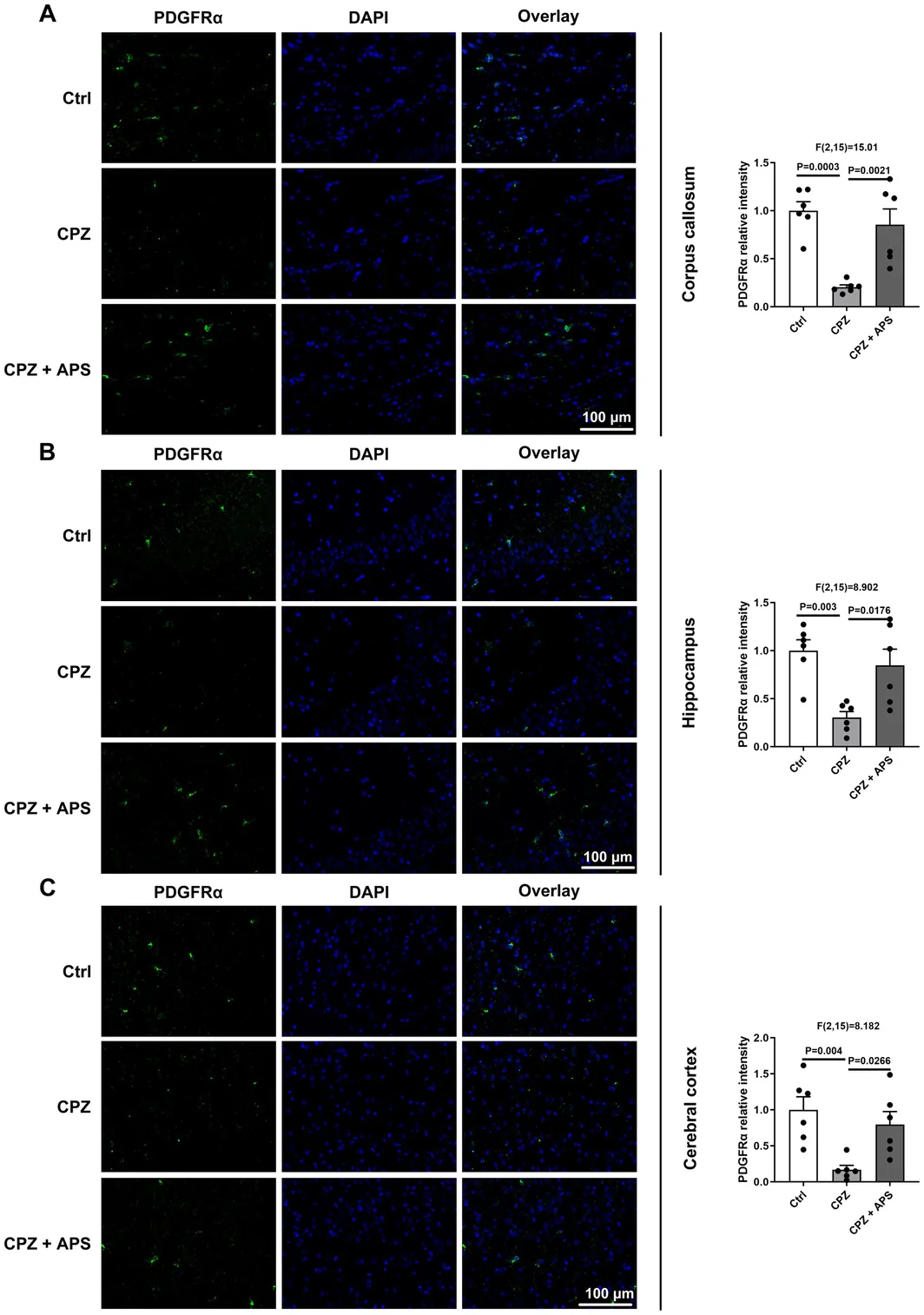
Pretreatment with astragalus polysaccharide inhibited the reduction of oligodendrocyte precursors in the demyelination model. (A–C) Immunofluorescence image analysis and statistical analysis of PDGFRα expression in the corpus callosum, hippocampus, and cerebral cortex were performed in different groups of mice. Scale bar = 100 μm. Statistical results are illustrated as mean ± SEM, *n* = 6. PDGFRα, platelet‐derived growth factor receptor alpha.

### 3.3 Astragalus Polysaccharide Alleviates MBP Loss and Apoptosis in the CPZ‐Induced Demyelination Model

We further detected the expression levels of MBP, which represents a major constituent of the myelin sheath, by immunohistochemistry and Western blotting. Immunohistochemical observations showed that MBP expression was significantly lower in the corpus callosum, hippocampus, and cortex of CPZ‐treated mice compared to controls, whereas APS treatment increased MBP levels (all *p* < 0.001, Figure 3A). Western blotting confirmed these findings. In comparison with the blank control cohort, MBP protein expression was notably lower in the CPZ‐treated group, and this reduction was reversed by APS treatment (all *p* < 0.001, Figure 3B,C). The expression levels of apoptosis‐associated proteins including Bax and Cleaved‐caspase 3 were further detected via Western blot analysis. As demonstrated in Figures 3B,D,E, Bax and Cleaved‐caspase 3 protein levels were considerably higher in the CPZ group than in the control group (*p* < 0.001, *p* < 0.01), whereas APS treatment suppressed this increase (*p* < 0.001). These results indicate that APS treatment ameliorates MBP loss and reduces apoptosis in the CPZ‐induced demyelination model. Collectively, these findings suggest that APS exerts a myelin‐protective effect by attenuating myelin loss in demyelination models.

**FIGURE 3:**
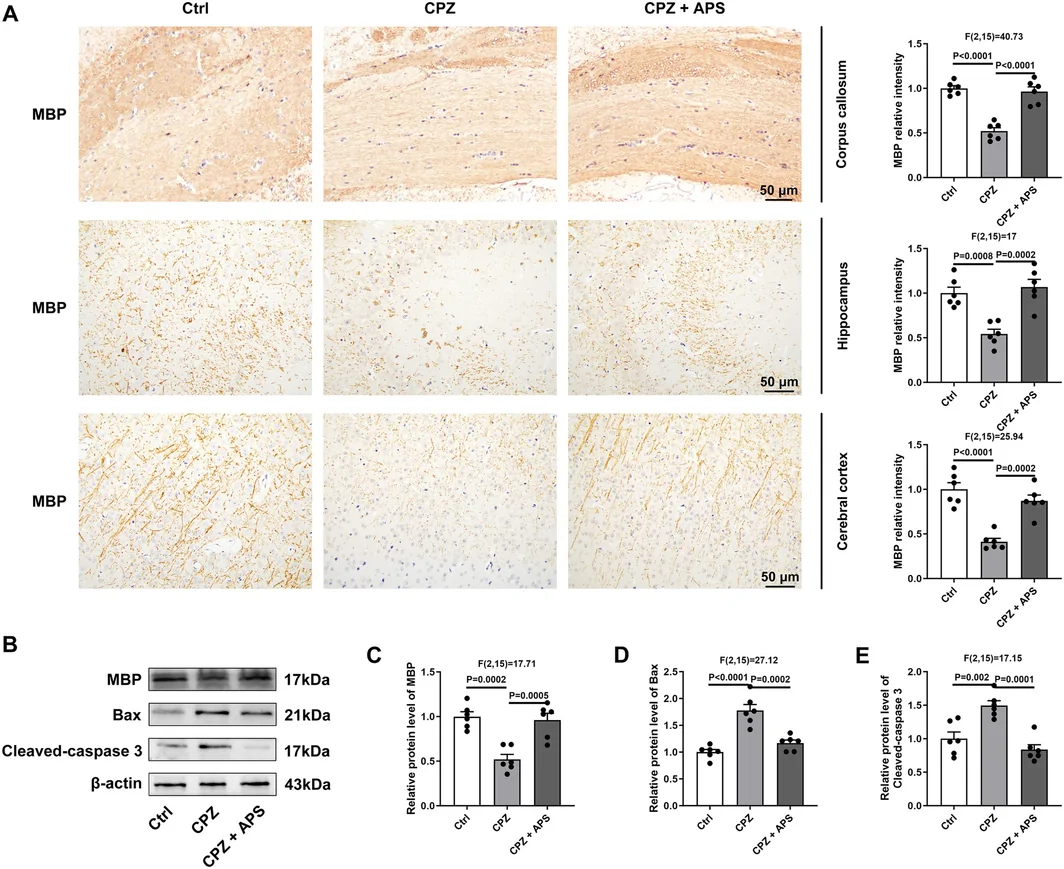
Astragalus polysaccharide alleviated MBP reduction and apoptosis in the demyelination model. (A) Representative immunohistochemical images demonstrating the relative intensity and expression of MBP in the corpus callosum, hippocampus, and cerebral cortex. Scale bar = 50 μm. (B–E) Western blot images representative of MBP, Bax, and cleaved‐caspase 3 protein expression in the brains of the three groups of mice and corresponding statistical analysis. Statistical results are illustrated as mean ± SEM, *n* = 6. MBP, myelin basic protein.

### 3.4 Astragalus Polysaccharide Alleviates Oxidative Stress in the CPZ‐Induced Demyelination Model

We assessed the expression levels of target proteins Nrf2 along with HO‐1, key markers of oxidative stress, by immunohistochemistry and Western blotting. Immunohistochemical analysis found the CPZ group's Nrf2 expression was substantially lower than that of the controls (*p* < 0.001, Figure 4A), whereas HO‐1 expression was markedly increased (*p* < 0.001, Figure 4A). In contrast, APS treatment upregulated Nrf2 and downregulated HO‐1 expression (all *p* < 0.001, Figure 4A). Western blotting confirmed these findings, demonstrating that intervention with APS could reverse the downregulation of Nrf2 (all *p* < 0.01, Figure 4B,C) and the upregulation of HO‐1 induced by CPZ (all *p* < 0.001, Figure 4B,D). Based on these results, it may be deduced that APS attenuates the oxidative stress response in the CPZ‐induced demyelination model.

**FIGURE 4:**
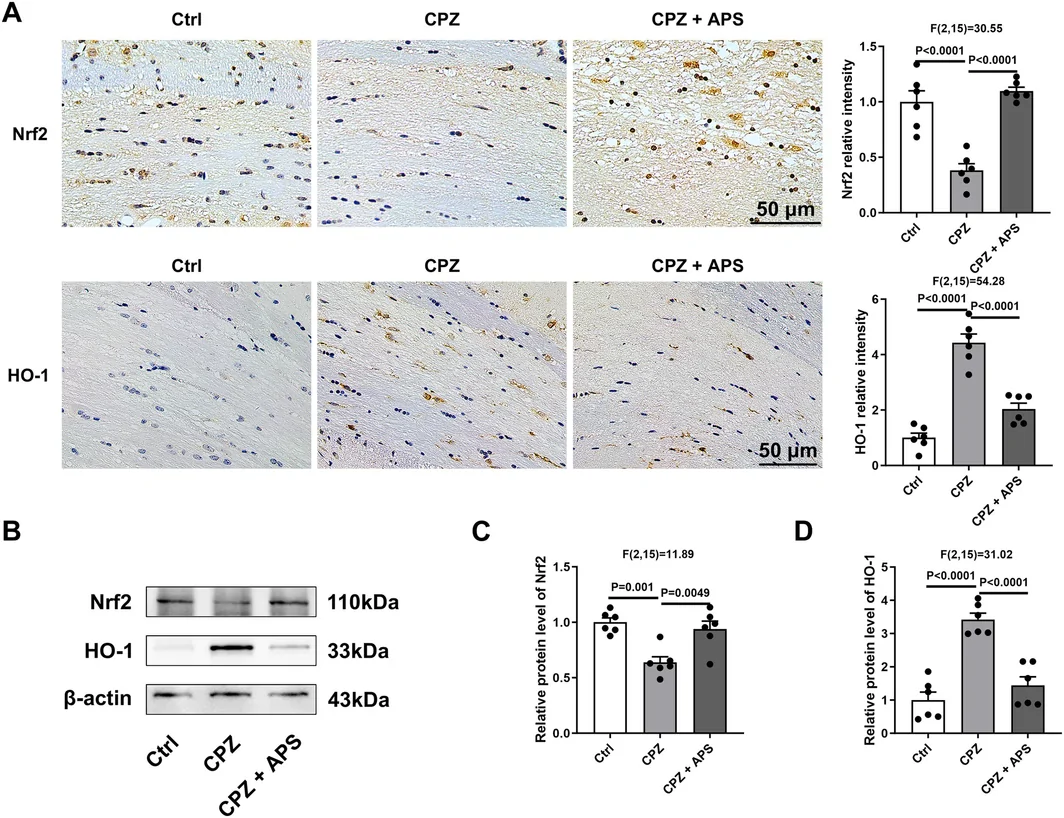
Astragalus polysaccharide alleviates oxidative stress in the demyelination model. (A) Representative immunohistochemical images presenting the relative intensity and expression of Nrf2 and HO‐1 in the corpus callosum. Scale bar = 50 μm. (B–D) Western blot bands of HO‐1 and Nrf2 protein expression in the brains of the three groups of mice and corresponding statistical analysis. Statistical results are illustrated as mean ± SEM, *n* = 6. HO‐1, heme oxygenase‐1; Nrf2, nuclear factor erythroid 2‐related factor 2.

### 3.5 Astragalus Polysaccharide Reduces H_2_O_2_ ‐Induced Apoptosis and ROS Accumulation in Oligodendrocytes

An in vitro oxidative stress model was established by exposing OLN93 cells to H_2_O_2_. As shown in Figure 5A, the CCK‐8 assay revealed that cellular survival rates were approximately 50% subsequent to intervention using 400 μM H_2_O_2_ compared to the control group (*p* < 0.001). Therefore, 400 μM H_2_O_2_ was adopted in the follow‐up experimental investigations. The protective effect of various concentrations of APS against H_2_O_2_‐induced cytotoxicity was assessed utilizing the CCK‐8 test. As depicted in Figure 5B, APS exerted a concentration‐dependent protective effect. Specifically, treatment with 25 μg/mL APS gave rise to a significant elevation in cell viability (*p* < 0.01). Therefore, subsequent experiments were performed with 25 μg/mL APS.

**FIGURE 5:**
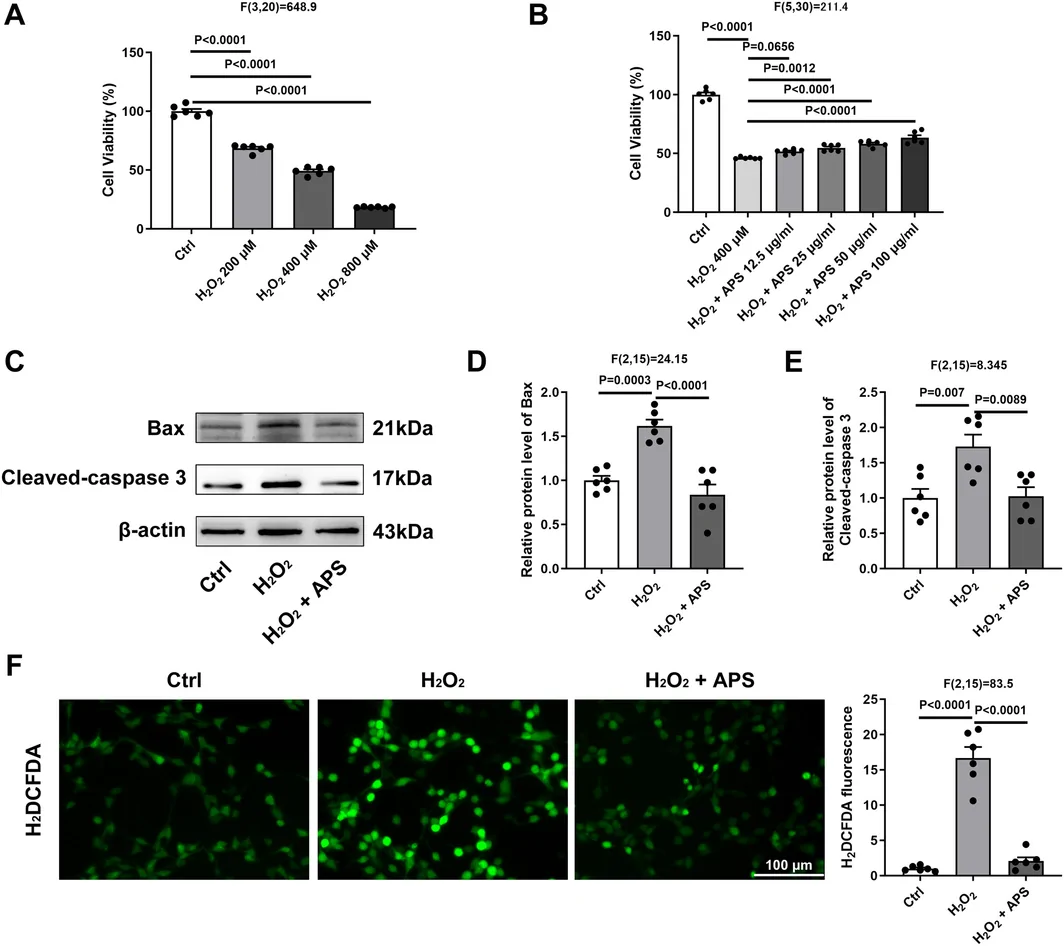
Astragalus polysaccharide reduces H_2_O_2_‐induced oligodendrocyte apoptosis and oxidative stress. (A) Influence of different dosages of H_2_O_2_ on oligodendrocyte viability (*n* = 6). (B) Influence of different APS concentrations on cell survival in H_2_O_2_‐induced oligodendrocyte death (*n* = 6). (C–E) Representative Western blot images of Bax and cleaved‐caspase 3 protein levels in H_2_O_2_‐induced oligodendrocyte death and corresponding statistical analyses. (F) Representative imaging and statistical data assessing ROS expression in the cell culture model are shown (*n* = 6). Scale bar = 100 μm. Statistical results are illustrated as mean ± SEM, *n* = 6. H_2_O_2_, hydrogen peroxide; ROS, reactive oxygen species.

Western blot assays were conducted to examine the abundance of Bax and Cleaved‐caspase 3, two apoptosis‐related proteins. As illustrated in Figure 5C–E, a pronounced rise in Bax and Cleaved‐caspase 3 protein levels occurred in response to H_2_O_2_ (*p* < 0.001, *p* < 0.01), relative to the control group, whereas APS pretreatment attenuated these increases (*p* < 0.001, *p* < 0.01). Furthermore, APS significantly inhibited H_2_O_2_‐induced ROS accumulation. As shown in Figure 5F, when compared to controls, H_2_O_2_‐treated cells had noticeably higher ROS levels (*p* < 0.001); however, APS treatment considerably decreased this rise (*p* < 0.001). These outcomes reveal that APS inhibits H_2_O_2_‐induced apoptosis and ROS accumulation in oligodendrocytes.

### 3.6 Astragalus Polysaccharide Alleviates H_2_O_2_ ‐Induced Oxidative Stress in Oligodendrocytes

To further evaluate HO‐1 and Nrf2 expression, Western blotting and fluorescence immunostaining assays were conducted simultaneously. As illustrated in Figure 6A–D, the protein level of Nrf2 was markedly reduced within the H_2_O_2_‐treated cells compared to controls (all *p* < 0.001), whereas APS treatment restored its expression (*p* < 0.001, *p* < 0.01). Conversely, exposure to H_2_O_2_ triggered a prominent elevation in HO‐1 protein levels (all *p* < 0.001), and this increase was attenuated by APS treatment (*p* < 0.001, *p* < 0.01, Figure 7B–D). These in vitro data are in accordance with the corresponding in vivo findings, showing that APS affords defense against oligodendrocyte damage via the modulation of oxidative stress along with the reduction of apoptosis.

**FIGURE 6:**
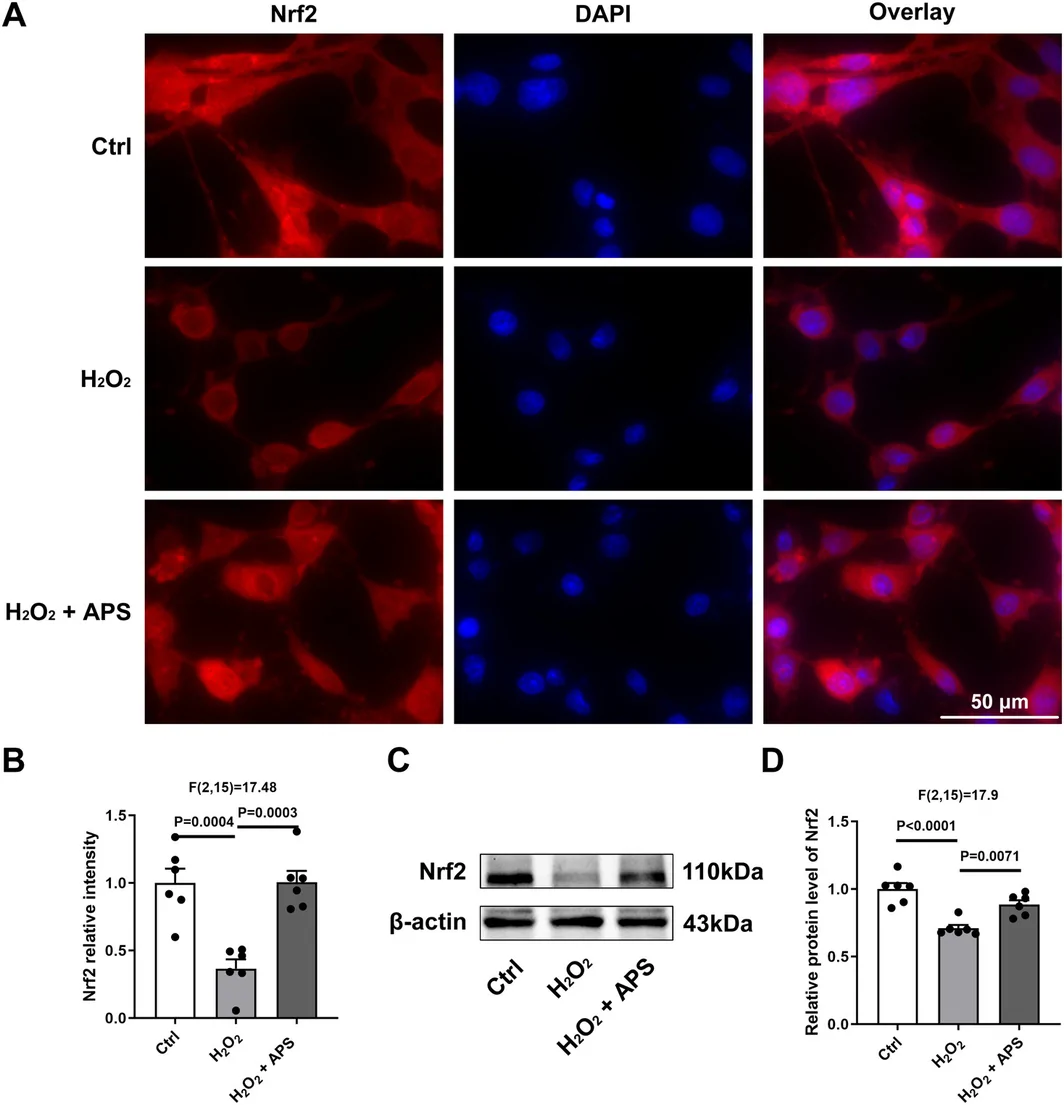
Astragalus polysaccharide alleviated H_2_O_2_‐induced oxidative stress in oligodendrocytes. (A, B) Typical immunofluorescence micrographs and statistical analysis of Nrf2 expression in H_2_O_2_‐induced oligodendrocyte death. Scale bar = 50 μm. (C, D) Typical Western blot bands and statistical analysis of Nrf2 protein levels in H_2_O_2_‐induced oligodendrocyte death. Statistical results are illustrated as mean ± SEM, *n* = 6.

**FIGURE 7:**
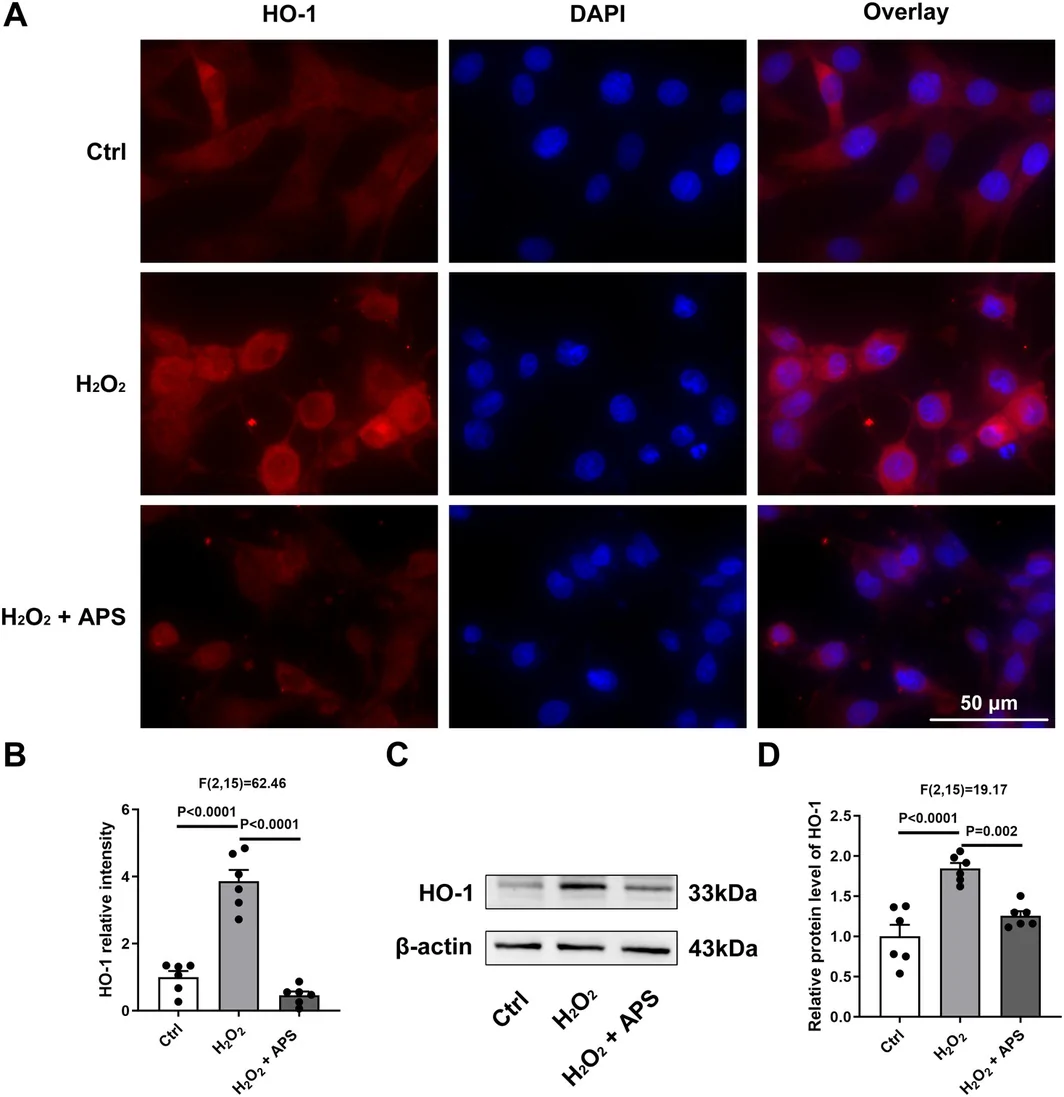
Astragalus polysaccharide alleviated H_2_O_2_‐induced oxidative stress in oligodendrocytes. (A, B) Typical immunofluorescence micrographs along with statistical analysis of HO‐1 expression levels in H_2_O_2_‐induced oligodendrocyte death. Scale bar = 50 μm. (C, D) Shown are representative Western blot results together with statistical evaluation of HO‐1 protein levels in H_2_O_2_‐induced oligodendrocyte death. Statistical results are illustrated as mean ± SEM (*n* = 6).

### 3.7 Inhibition of Nrf2 Abolishes the Antioxidant Effect of APS on H_2_O_2_ ‐Induced Injury in Oligodendrocytes

Having established that APS restores Nrf2 and suppresses HO‐1 in H_2_O_2_‐exposed OLN93 cells, we then investigated whether the protective effect of APS depends on Nrf2 activation. OLN93 cells were therefore challenged with H_2_O_2_ and treated with APS in the presence or absence of the selective Nrf2 inhibitor ML385. Both immunofluorescence staining and Western blotting revealed that the APS‐mediated elevation of Nrf2 was largely abolished by ML385 co‐treatment, as reflected by the weaker Nrf2 fluorescence intensity and the decreased Nrf2 protein levels relative to the H_2_O_2_ + APS group (all *p* < 0.05, Figure 8A–D). In parallel, the APS‐driven decline in HO‐1 was significantly offset once Nrf2 was blocked, with HO‐1 fluorescence intensity and protein levels rising again toward those seen in H_2_O_2_‐treated cells (*p* < 0.001, *p* < 0.05, Figure 8E–H). Because HO‐1 induction in this setting represents an adaptive response to the oxidative burden rather than a simple readout of Nrf2 transcriptional activity, this rebound is consistent with a reinstatement of oxidative stress upon Nrf2 inhibition. Corroborating this interpretation, H_2_DCFDA staining showed that ML385 markedly compromised APS's ROS‐scavenging capacity, so ROS levels in the H_2_O_2_ + APS + ML385 group were significantly higher than in the H_2_O_2_ + APS group (*p* < 0.001, Figure 8I,J). Taken together, these data indicate that the antioxidant effects of APS in oligodendrocytes depend largely on the Nrf2 signaling pathway and that the protective effects of APS against H_2_O_2_‐induced oxidative damage are based on the activation of the Nrf2/HO‐1 axis, rather than on the isolated regulation of any single component of this axis.

**FIGURE 8:**
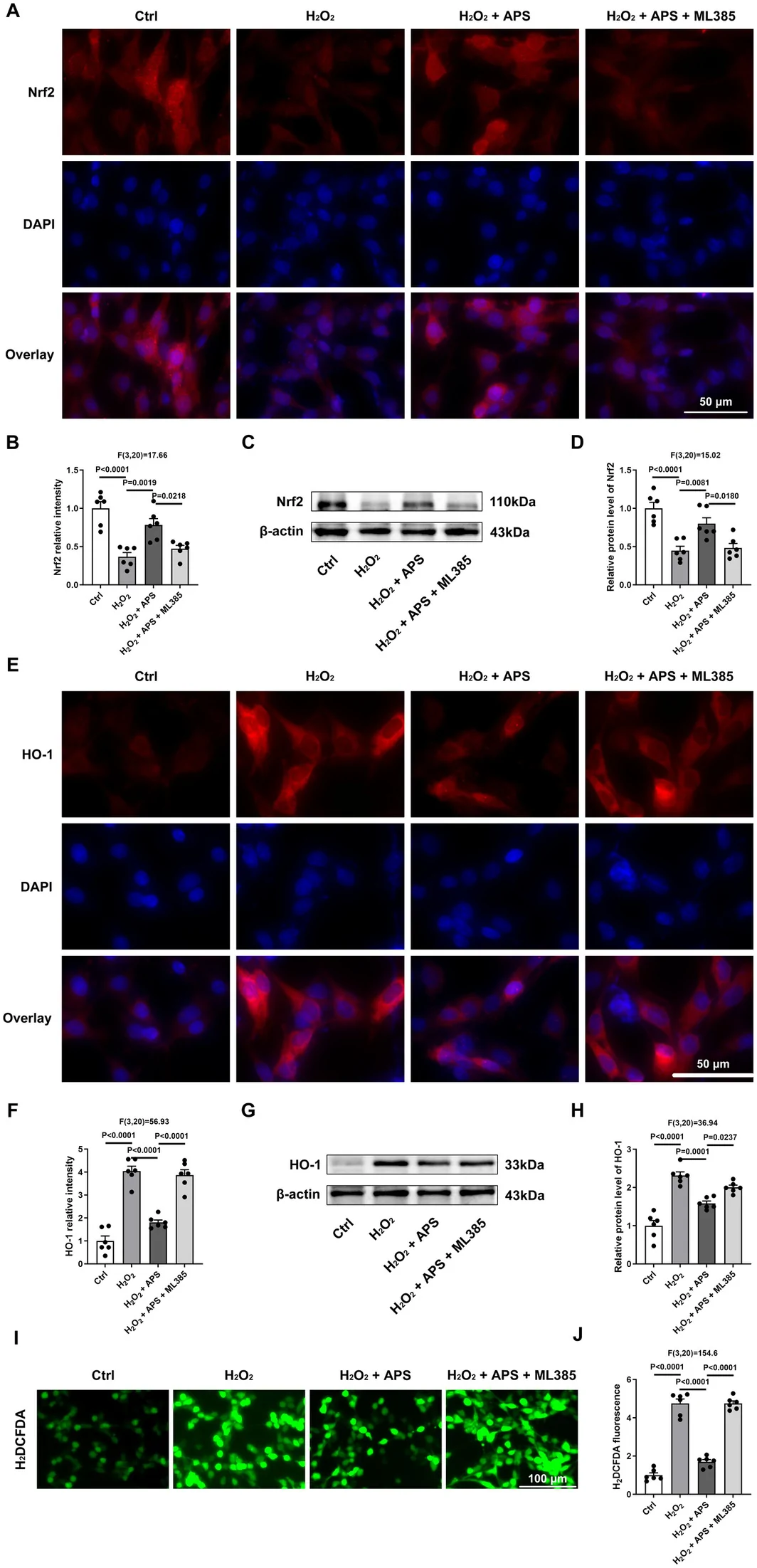
APS protects OLN93 cells against H_2_O_2_‐induced oxidative stress in an Nrf2‐dependent manner. (A–D) Nrf2 expression assessed by immunofluorescence staining and Western blotting, with corresponding quantification. (E–H) HO‐1 expression assessed by immunofluorescence staining and Western blotting, with corresponding quantification. (I, J) Representative H_2_DCFDA fluorescence images and quantification of intracellular ROS levels. Scale bar = 50 μm (A, E) and 100 μm (I). Data are presented as the mean ± SEM (*n* = 6).

## 4 Discussion

In the CNS, the myelin sheath is a multilayered membrane structure formed by oligodendrocyte processes that wrap around neuronal axons. The myelin sheath plays an essential role in electrical insulation, accelerating nerve impulse conduction, reducing axonal energy consumption, providing trophic support, and maintaining long‐term axonal integrity (Bercury and Macklin 2015; Fields 2014; Fünfschilling et al. 2012; Nave 2010a; Nave 2010b). However, oligodendrocytes and myelin are highly susceptible to injury from various insults, including infection, toxin exposure, and degenerative or autoimmune conditions (Merrill and Scolding 1999). Myelin abnormalities can result in axonal dysfunction, significant pathological changes, and neurological deficits, as observed in various nervous system disorders (Goldstein et al. 2016). Among these, MS stands as the most prevalent demyelinating disease of the CNS (Merrill and Scolding 1999). Therefore, the development of therapeutic agents that protect the myelin sheath and oligodendrocytes is crucial for the treatment of MS.

We employed a CPZ‐induced demyelination model and assessed oligodendrocyte expression. Fluorescent immunostaining detection demonstrated that the relative level of the oligodendrocyte‐specific biomarker Olig2 decreased markedly within the modeled group when compared with the normal control group, whereas APS treatment markedly restored its expression. Mature myelinating oligodendrocytes are derived from OPCs (Xiao and Czopka 2023). Our results demonstrated that APS prevented the loss of OPCs in CPZ‐induced demyelinating mice. Furthermore, APS significantly attenuated the decrease in MBP and suppressed the elevated expression of pro‐apoptotic biomarkers, including cleaved‐caspase 3 and Bax. These findings indicate that APS effectively mitigates myelin damage and apoptosis in the CPZ‐induced demyelination mouse model.

Oxidative stress has been increasingly recognized as a critical contributor to MS pathogenesis (Jiménez‐Jiménez et al. 2024). The transcription factor Nrf2 serves as a master regulator of cellular antioxidant defenses (Chen et al. 2015). Under basal conditions, Nrf2 is sequestered in the cytoplasm by its repressor Keap1, which targets it for ubiquitin‐proteasomal degradation (O'Rourke et al. 2024). Upon oxidative or electrophilic stress, specific cysteine residues on Keap1 undergo conformational modification, releasing Nrf2 to translocate into the nucleus, where it heterodimerizes with small Maf proteins and binds to the antioxidant response element (ARE) (O'Rourke et al. 2024). This initiates the transcription of a battery of cytoprotective genes, including GCL, NQO1, GSTs, and HO‐1 (Kansanen et al. 2013; Baird and Yamamoto 2020). Among these, HO‐1 is markedly upregulated in response to oxidative stress, including exposure to H_2_O_2_ (Loboda et al. 2016). Consistency exists between these findings and the data obtained from our experiments. In the CPZ‐induced demyelination model, the expression of Nrf2 was substantially decreased, while a significant elevation of HO‐1 expression occurred, indicating the presence of oxidative stress. Notably, APS treatment significantly upregulated Nrf2 expression, suppressed HO‐1 overexpression, and restored redox homeostasis. In vitro, APS treatment markedly inhibited H_2_O_2_‐induced apoptosis and ROS accumulation. Concurrently, APS treatment upregulated Nrf2 protein levels and dampened the upsurge in HO‐1. Collectively, these data support the notion that Nrf2 activation reinforces the antioxidant status of cells and participates in the neuroprotective effects exerted by APS.

Polysaccharides, as a class of compounds, generally do not possess the strong hydrogen‐donor chemical properties of polyphenols and small‐molecule antioxidants. Their reported antioxidant activity is usually achieved through cellular signal transduction rather than direct chemical free radical scavenging (Chen et al. 2026). The reduction of intracellular reactive oxygen species and the regulation of Nrf2 and HO‐1 are more reasonably explained as an antioxidant response mediated by signals. It is notable that the functional loss experiment using the selective Nrf2 inhibitor ML385 confirmed that the antioxidant and neuroprotective effects of APS are largely dependent on the functional Nrf2 signaling pathway: when ML385 is used simultaneously, the Nrf2 upregulation induced by APS is eliminated, the upregulation of HO‐1 is counteracted, and the ROS scavenging ability of OLN93 cells is significantly weakened (Figure 8). These findings indicate that APS is not a direct free radical scavenger, but may be a signaling molecule that activates the transcription program regulated by Nrf2, thereby coordinating the regulation of a series of cell‐protecting genes (such as HO‐1), and jointly enhancing the cell's resistance to oxidative damage.

At the same time, APS has been proven to have anti‐inflammatory and immunomodulatory effects. APS can inhibit microglial activation, suppress the TLR4/NF‐κB signaling pathway, downregulate pro‐inflammatory cytokines, and regulate macrophage M1/M2 polarization (Guo et al. 2023; Yu et al. 2019). This suggests that the partial oxidative stress observed in vivo may be due to the weakened activation of microglial cells and resident immune signaling pathways. Activated NF‐κB competes with Nrf2 for the limited transcriptional co‐activator CBP/p300, thereby promoting Keap1 expression and inhibiting the transcription driven by the antioxidant response element; while the activation of Nrf2 and its downstream effector HO‐1 can inhibit NF‐κB‐mediated inflammatory transcription through the bilirubin and iron metabolism pathways (Wardyn et al. 2015; Cerdán‐Centeno et al. 2026; Soares et al. 2004). Moreover, in demyelinating injury sites, oxidative stress and NF‐κB‐mediated inflammation also have an enhancing interaction (Yuan et al. 2026). These interactions indicate that the changes in the Nrf2 signaling pathway observed and the anti‐inflammatory effect of APS are not mutually exclusive. The reduction of oxidative damage may be due to the weakening of the inflammatory signaling pathways. Therefore, we interpret the protective effect of APS as a comprehensive antioxidant and anti‐inflammatory effect, where the Nrf2/HO‐1 axis is the core but not the only component, and we believe that directly detecting this integrated mechanism in the cuprizone model is an important task for future research.

Several mechanisms by which systemically administered APS reaches the CNS and produces neuroprotective actions have yet to be elucidated. Because APS is a hydrophilic polysaccharide with a high molecular weight, it penetrates the intact BBB only with difficulty. Notably, following administration, APS can undergo hydrolysis to yield low‐molecular‐weight oligosaccharides (Cai et al. 2023). Furthermore, in mice subjected to cuprizone‐induced demyelination, early‐stage BBB permeability has been reported to increase (Berghoff et al. 2017), a condition that would favor the transit of such oligosaccharides into the CNS, where they could mediate neuroprotection. At present, these ideas remain hypothetical and require experimental confirmation.

Nevertheless, adequate evidence now supports the view that APS can produce neuroprotection via indirect routes. First, through systemic immunomodulation, APS exhibits strong anti‐inflammatory and immune‐regulatory actions, which may lessen peripheral inflammation and consequently lower the inflammatory load on the CNS (Li et al. 2022). Second, via gut‐brain axis regulation, emerging data indicate that polysaccharides can reshape the gut microbiota, thereby affecting neural activity through immune and endocrine (Li et al. 2026). Indeed, recent work has demonstrated that APS preserves BBB integrity in pathological settings, potentially allowing other protective molecules to access the CNS (Yao et al. 2026).

We acknowledge, however, that our present investigation does not fully reconcile in vivo systemic delivery with in vitro direct cell treatment, and this constitutes a limitation. Intraperitoneally administered APS must undergo ADME processes and BBB penetration before reaching oligodendrocytes, whereas in vitro experiments applied APS directly to OLN93 cells at defined concentrations without pharmacokinetic barriers or multicellular crosstalk. Moreover, the repeated i.p. dosing over weeks in vivo differs markedly from acute 24 h pretreatment in vitro—a distinction of particular relevance for the time‐dependent Nrf2 transcriptional program (Cuadrado et al. 2019). Future experiments involving direct CNS delivery (e.g., intraventricular injection) or assessment of BBB permeability will help validate the proposed in vivo mechanism of APS.

Collectively, this study demonstrates that APS attenuates CPZ‐induced demyelination and oligodendrocyte injury. Furthermore, we demonstrate through experiments conducted in vivo alongside those performed in vitro, APS confers neuroprotection through activation of the Nrf2/HO‐1 pathway and suppression of oxidative stress. Evidence presented in this work supports APS as a potential therapeutic candidate for multiple sclerosis, while also providing a pharmacological rationale for exploring innovative therapeutic strategies.

## Author Contributions

**Yijiao Long:** data curation, conceptualization, investigation, methodology. **Lihui Wang:** methodology, conceptualization. **Baohua Hou:** conceptualization, investigation, software, data curation.

## Funding

This work was financially supported by the National Natural Science Foundation of China (grant no. 32300803), the Doctoral Fund Project of Henan Polytechnic University (B2021‐68).

## Ethics Statement

All experimental procedures complied with the Medical Research Ethics Committee of Henan Polytechnic University (Permit Number: HPU2024‐104).

## Conflicts of Interest

The authors declare no conflicts of interest.

## Data Availability

The data that support the findings of this study are available from the corresponding author upon reasonable request.
